# Bayesian probabilistic network modeling from multiple independent replicates

**DOI:** 10.1186/1471-2105-13-S9-S6

**Published:** 2012-06-11

**Authors:** Kristopher L Patton, David J John, James L Norris

**Affiliations:** 1 Department of Mathematics, Wake Forest University, Winston-Salem, North Carolina 27109, USA; 2 Department of Computer Science, Wake Forest University, Winston-Salem, North Carolina 27109, USA

## Abstract

Often protein (or gene) time-course data are collected for multiple replicates. Each replicate generally has sparse data with the number of time points being less than the number of proteins. Usually each replicate is modeled separately. However, here all the information in each of the replicates is used to make a composite inference about signal networks. The composite inference comes from combining well structured Bayesian probabilistic modeling with a multi-faceted Markov Chain Monte Carlo algorithm. Based on simulations which investigate many different types of network interactions and experimental variabilities, the composite examination uncovers many important relationships within the networks. In particular, when the edge's partial correlation between two proteins is at least moderate, then the composite's posterior probability is large.

## Introduction

Often the laboratory collection of protein phosphorylation time-course data results not in a single set of time-course data, but in multiple sets of time-course data. Typically the data are sparse: the number *t *of time points is significantly less than the number *k *of proteins. Even though there are differences between these data sets, the underlying biochemical interactions (signal) are reflected in each of these data sets. Many times these individual sets of time course protein data are modeled individually. The discussion in this paper focuses on protein measurements. However, it equally applies to sparse time course measurements obtained through gene microarrays.

The methods in this paper incorporate data from multiple replications of a systems biology investigation to determine composite posterior probabilities of network relationships. These methods are motivated by the desire to predict interactions between proteins based on probabilistically incorporating all of the data from several independent investigations. Utilizing underlying Gaussian-based regression likelihood, low informative empirical priors and Bayesian model averaging, closed form (up to a proportional constant) posterior probabilities are computed of networks, each of which is a directed acyclic graph (DAG). Extensive searching through the space of DAGs is performed with a multi structured Metropolis-Hastings Markov Chain Monte Carlo based algorithm. These model DAG posterior probabilities are combined with Bayesian model averaging [1] to produce posterior probabilities for relationships between the proteins.

Since the combined likelihood from our *m *independent replications has approximately *m *times more information, whether Akaike, Bayesian, Dirichlet information criteria or Fisher information, than that of a single replication, the combined technique tends to yield more precise estimates for posterior probabilities [2,3]. Also in this paper, simulations demonstrate that this combined analysis captures more of a network's signal.

In a previous paper [4], an approximate Bayesian posterior analysis for a single sparse replication was developed. As with the current paper, it used multiple regression to model cotemporal associations between the proteins' measurements, where each sampled time provided insight into the proteins' relationships. Diagnostics to test the suitability of this [4] method for a particular data set were presented. These diagnostics easily can be employed on each separate replication of a multiple replication study in order to test the current paper's suitability for a particular multiple replication study. Furthermore, many of the theoretical justifications from [4] carry over to the multiple replication setting. In particular, the previous and current methodology strongly relates to low-order (small number of predictors) dependency networks [5-7]. The use of DAGs, with the proteins being the nodes and directed edges signifying the relations, allows the splitting of a replicates' likelihood into conditionally independent parts [6,8-10]. While in [4] approximate posterior probabilities were obtained for a single replication through the use of Bayesian information based scores, in the current paper an exact Bayesian posterior probability is obtained for the combined *m *replications.

Recent papers have examined combining data from multiple studies. In [11], a score function based on the expected number of associations was developed, and its results were weighted in a Bayesian fashion with supplementary information from gene ontology and protein structures. In a frequentist, non-Bayesian manner, the authors in [12] weighted different studies so as to maximize the statistical power (chance of claiming a true positive). They also obtained integrated p-value estimates. In [13], linear programming was used to find the subnetworks which are most consistent from one replication to the next.

## Methods

Edge probabilities are computed for an undirected graph where the nodes represent individual proteins and where an edge between two nodes represents a relationship between the two corresponding proteins. These edge probabilities are based on an algorithmic search through the space of all models (DAGs) guided by the posterior probability of the DAGs. This DAG posterior probability takes into account all of the data sets. Verification of the effectiveness of this technique requires simulations. A simulation consists of generating multiple sets of data from the same underlying signal. The following discussions focus on each of the following important ideas: DAG posterior probability, algorithmic search, testing data sets and analysis techniques.

### Posterior probability

Our mathematical space of network models consists of directed acyclic graphs. The vertices represent proteins and the directed edges signify *parents-child *linear associations between the proteins. In particular, the set of parents (predictors) of a particular child (response) is the set of vertices which have directed edges going from the parent to the child. In order to give equal consideration to each child and each potential parent, each protein's time course values within a data set are standardized, using its average and standard deviation. The number of parents for any particular child is restricted to be less than or equal to *t - *2, guaranteeing valid regression settings [14]. Acyclic refers to not allowing cycles in the graph, i.e. not allowing a protein to be a direct descendent of itself. In this paper, we present a theoretically strong probabilistic method which comprehensively incorporates multiple data sets. For convenience, the data sets are referred to as *reps*, even though the reps may have different signal parameters and may differ in their variances about the underlying signal network. Separately for each DAG, rep and child combination, we utilize independent unit-informative empirical g-priors for the slope parameters of the parents-child linear regression relations that are specified by the DAG [15]. As well, independent unit informative inverse gamma priors are independently placed on the residual variances. Thus, due to the prior structure, the reps' data sets are independent from one replicate to the next. In addition, due to the DAG structure, each child's conditional likelihood is independent from that of another child [8]. Therefore a particular DAG's Bayes factor, is

(1) $$P_{r}\left(Y|DAG\right)=\underset{\underset{i}{\text{children}}}{\prod }\underset{\underset{j}{\text{reps}}}{\prod }P_{r}\left(Y_{i}^{\left(j\right)}|DAG\right)$$

where *Y *represents the (standardized) data, and $Y_{i}^{\left(j\right)}$is the data for child *i *in rep *j*. Due to the conjugacy nature of the priors, the child *i *rep *j *Bayes factor, $P_{r}\left(Y_{i}^{\left(j\right)}|DAG\right)$, has a closed form expression [15, Chapter 9]. Specifically, this Bayes factor is given by

(2) $$P_{r}\left(Y_{i}^{\left(j\right)}|DAG\right)=\left\{\begin{array}{cc} \pi^{t/2}\frac{\Gamma \left(\left(1+t\right)/2\right)}{\Gamma \left(1/2\right)}{{\left(1+t\right)}^{-P}}^{\left(i\right)/2}\frac{s_{ij}^{2}}{{\left(s_{ij}^{2}+SSR_{i}^{\left(j\right)}\right)}^{\left(1+t\right)/2}} & \text{if}\;P\left(i\right)\geq 1\\ {\left(2\pi \right)}^{-t/2}e^{-\left(t-1\right)/2} & \text{if}\;P\left(i\right)=0 \end{array}\right)$$

where *P*(*i*) is the number of parents of child *i *for the DAG, $X_{i}^{\left(j\right)}$is child *i*'s parents data matrix for rep *j *for the DAG, $s_{ij}^{2}$ is the corresponding residual regression (error) variance, and

$$SSR_{i}^{\left(j\right)}={\left(Y_{i}^{\left(j\right)}\right)}^{T}\left(I-\frac{t}{t+1}X_{i}^{\left(j\right)}{\left({\left(X_{i}^{\left(j\right)}\right)}^{T}X_{i}^{\left(j\right)}\right)}^{-1}{\left(X_{i}^{\left(j\right)}\right)}^{T}\right)Y_{i}^{\left(j\right)}.$$

The simple value of $P_{r}\left(Y_{i}^{\left(j\right)}|DAG\right)$when *i *has no parents is a consequence of the standardized data.

From Equations (1) and (2), a closed form is easily found for the DAG's overall Bayes factor. As is common, we assume that the prior probability of one model (DAG) is the same as that of another. This yields that the posterior probability of a particular DAG, given all of the data, is proportional to the DAG's (overall) Bayes factor. Since for even a moderate number of proteins the DAG space is too large for a census, an intelligent search algorithm must be used.

### Algorithmic search

In a Markov chain manner, the Metropolis-Hastings algorithm moves through the DAG space. From equations (1) and (2), for any element, a DAG, its Bayes factor (proportional posterior probability) can be computed. Given a current element in the search space, the algorithm generates a candidate element from the current one. If the probability of the candidate is greater than the probability of the current element, then the candidate replaces the current element. If the probability of the candidate is not an improvement over the probability of the current element, then the candidate replaces the current element with a probability of $\frac{candidateBayesProb}{currentBayesProb}$. For each of 10 runs there are 50 million iterations, and the highest 200 scoring DAGs, along with their Bayes factors, are collected. These 10 lists of 200 are amalgamated into one list, *TopD*. From the list, *TopD*, probabilities for undirected graphs (*TopU*), protein-to-protein edge posterior probabilities, and other network feature probabilities are computed. Details of the Metropolis-Hastings algorithm are found in [16].

The Metropolis-Hastings algorithm used in this research is a variation of the one presented in [4,17,18]. This paper's single rep algorithm is a pure Bayes posterior modification of the previous Bayesian information criterion (BIC) based approximation algorithm [4]. The multiple rep algorithm has similarities to the single rep algorithm, but it moves through the DAG space based on Bayes posterior probabilities after incorporating multiple reps, and it allows multiple edge insertions and deletions.

The use of a single move, a single insertion or deletion of an edge, in a Metropolis-Hastings search is common and is motivated by the Metropolis requirement that all moves be reversible with equal probability of a move and its inverse [16]. In the multiple rep algorithm, single, double, or triple reversible moves are allowed. Each vertex in the directed acyclic graphs has bounded in-degree (a maximum number of parents of a given child), typically 3. This condition must be enforced as well. The implementation of multiple moves is straightforward. First, the number of changes (1, 2 or 3) is chosen uniformly. Second, using this chosen number, either edge insertions or deletions are selected and applied yielding a candidate directed acyclic graph. Third, if the candidate directed acyclic graph is found to be infeasible then the process of choosing a candidate starts over.

### Testing data

In order to assess the quality of the models found by the multiple rep algorithm, it is necessary to engineer replicate data where the underlying signal is known. For this paper, five studies of multiple replicate data are generated from known underlying signals.

The simulated sets of data are sampled directly from multivariate normal distributions, hence no preprocessing transformations are needed. To generate the data for a particular rep of *t *time points and *k *proteins, we draw *t *independent samples from a *k*-dimensional multivariate normal distribution which has a mean vector of zeros and a selected generating covariance matrix, which provides the selected network signal. We use covariance matrices that are block diagonal with first-order autocorrelations [14, page 414] within the blocks (and zero correlations between blocks). Specifically, if proteins *p*_1_, *p*_2_, and *p*_3 _constitute a block of 3 correlated proteins, then the covariance (equivalent to signal Pearson correlation) block corresponding to them is of the form:

(3) $$\left[\begin{array}{ccc} 1.0 & \rho & \rho^{2}\\ \rho & 1.0 & \rho \\ \rho^{2} & \rho & 1.0 \end{array}\right]$$

where *ρ *is the Pearson correlation between *adjacent *proteins within the block. We say that the (triple) block of three vertices is correlated with intensity *ρ*.

One benefit of the block structures is that we obtain closed-form solutions for the generating partial correlations [14, page 160] between the proteins. For the triple block with associated covariance matrix (3), the partial correlation matrix is:

(4) $$\left[\begin{array}{ccc} 1.0 & \frac{\rho }{\sqrt{\rho^{2}+1}} & 0.0\\ \frac{\rho }{\sqrt{\rho^{2}+1}} & 1.0 & \frac{\rho }{\sqrt{\rho^{2}+1}}\\ 0.0 & \frac{\rho }{\sqrt{\rho^{2}+1}} & 1.0 \end{array}\right]$$

For a block of 4 proteins, the partial correlations between *p*_1 _and *p*_2 _and between *p*_3 _and *p*_4 _equal $\frac{\rho }{\sqrt{\rho^{2}+1}}$, while the partial correlation between *p*_2 _and *p*_3 _is $\frac{\rho }{\rho^{2}+1}$. All other pairs of the four proteins have a partial correlation of zero.

### Analysis of data and models

Our overall strategy is to conduct five illustrative simulation studies where each study consists of a set of three reps, each generated from a specific signal. Each of the five studies are designed to examine potentially different characteristics of biological networks. The three reps in a study mimic biological replicates. For each study, the multiple rep Bayesian Metropolis-Hastings algorithm is applied to all three replicates, giving the composite results. For comparison purposes, the single rep algorithm is applied to each of the three individual reps. Separately, for the composite and for each of the individual executions of the modeling algorithms, the matrix of protein-protein edge posterior probabilities and the vector of within-block connectivity probabilities are obtained.

Given a block of nodes, they are *connected *if given any pair of the nodes in the block, there exists a sequence of edges from the first node in the pair to the second node in the pair, where each edge is incident only with nodes in the block. The probability that a block of nodes, representing our proteins, is connected is estimated by the sum of the probabilities of the top undirected graphs in which those nodes are connected. Mathematically, this is:

$$\overset{NTopU}{\underset{i=1}{\sum }}X\left(v_{1},...,v_{n},TopU_{i}\right)Prob\left(TopU_{i}\right),$$

where the characteristic function χ(*v*_1_*, . . . , v_n_, G*) has the value 1 if and only if the vertices *v*_1_*, . . . , v_n _*are connected in the undirected graph *G*. The computation of the characteristic function χ () for 3 and 4 nodes is straightforward.

## Results

Specific results of the five simulation studies are presented. The discussion of the first study is more detailed than that of the remaining four since some of the details of all five studies are quite similar. Following the discussion of the individual studies, a further analysis of the posterior probabilities is presented.

### Individual studies

The first of five simulation studies is a set of three reps, $R_{1}^{\left(1\right)}$, $R_{2}^{\left(1\right)}$ and $R_{3}^{\left(1\right)}$. Each of these reps reflects *t *= 10 simulated measurements of twelve proteins, $p_{1}^{\left(1\right)},\ldots ,p_{12}^{\left(1\right)}$. Furthermore, the underlying generating covariance matrix has assigned high correlation intensity, *ρ *= 0.94, to each of the four blocks $\left\{p_{1}^{\left(1\right)},\ldots ,p_{3}^{\left(1\right)}\right\}$, $\left\{p_{4}^{\left(1\right)},\ldots ,p_{6}^{\left(1\right)}\right\}$, $\left\{p_{7}^{\left(1\right)},\ldots ,p_{8}^{\left(1\right)}\right\}$, and $\left\{p_{10}^{\left(1\right)},\ldots ,p_{12}^{\left(1\right)}\right\}$, as described in the Methods. The observed Pearson correlations in the reps are close to the Pearson correlations of their generator.

Table 1 shows that for the four blocks of signal correlated proteins, our model exhibits extremely high posterior connectivity probabilities. In all the reps, except for the blocks $\left\{p_{1}^{\left(1\right)},\ldots ,p_{3}^{\left(1\right)}\right\}$, and $\left\{p_{10}^{\left(1\right)},\ldots ,p_{12}^{\left(1\right)}\right\}$ in $R_{2}^{\left(1\right)}$, the triple connectivity probability of the highly correlated proteins is 1.0. In addition, the average of the triple connectivity probabilities over all false triples does not exceed 0.0641.

**Table 1:** Study 1 posterior probabilities

	C^(1)^	$R_{1}^{\left(1\right)}$	$R_{2}^{\left(1\right)}$	$R_{3}^{\left(1\right)}$
$p_{1}^{\left(1\right)}\;-p_{2}^{\left(1\right)}\;-p_{3}^{\left(1\right)}$	1.0	1.0	0.9744	1.0
$p_{4}^{\left(1\right)}\;-p_{5}^{\left(1\right)}\;-p_{6}^{\left(1\right)}$	1.0	1.0	1.0	1.0
$p_{7}^{\left(1\right)}\;-p_{8}^{\left(1\right)}\;-p_{9}^{\left(1\right)}$	1.0	1.0	1.0	1.0
$p_{10}^{\left(1\right)}\;-p_{11}^{\left(1\right)}\;-p_{12}^{\left(1\right)}$	1.0	1.0	0.993	1.0
TFP Avg	0.0376	0.0246	0.0641	0.0346
TLFP Avg	0.0008	0.0008	0.0047	0.0017

For the first study, the posterior connectivity probabilities for the four signal connected blocks are shown. For the composite model and the single rep models, all of these are quite high. The last two rows indicate the posterior probabilities (raw average and average via initial log odds transformation) over all false possible threesomes of vertices. These average posterior probabilities and average log odds are quite low.

**Figure 1:**
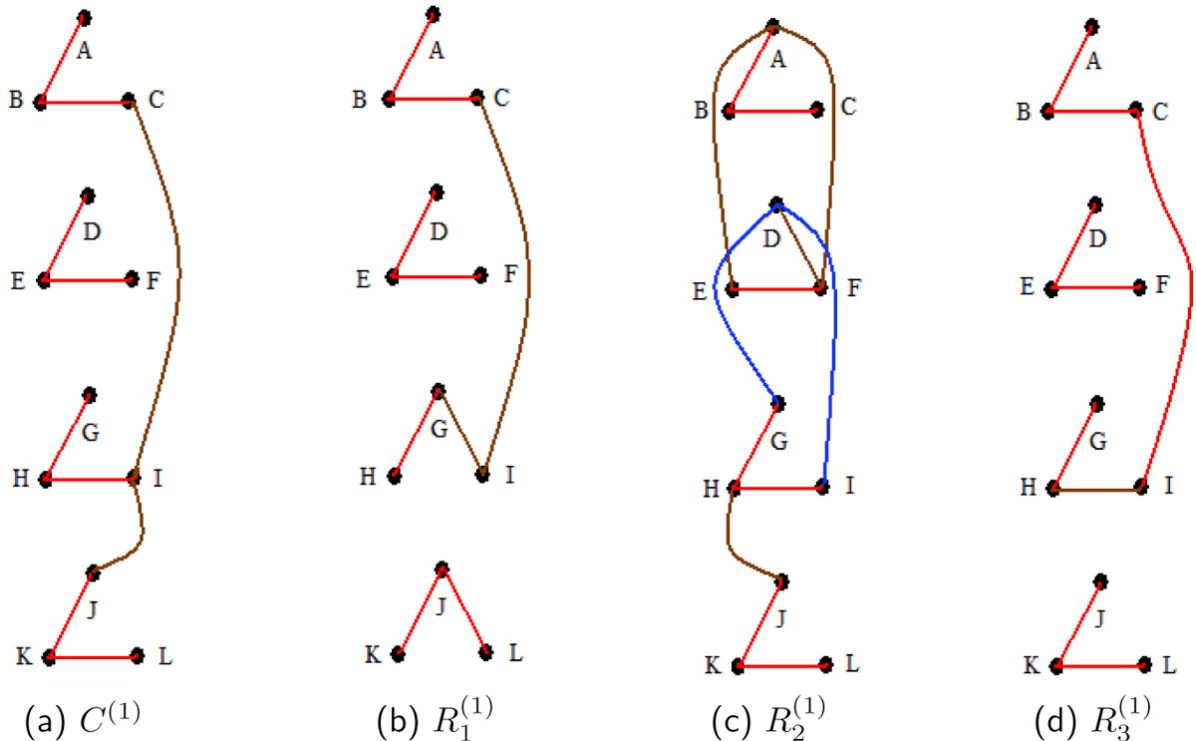
**Study 1 undirected edge posterior probabilities**. For the first study, (a), (b), (c) and (d) are representations of the undirected edge posterior probabilities. These are computed for the composite of all the replicates, *C*^(1)^, and for the individual replicates, $R_{1}^{\left(1\right)}$, $R_{2}^{\left(1\right)}$ and $R_{3}^{\left(1\right)}$. Blocks of vertices $\left\{p_{1}^{\left(1\right)},p_{2}^{\left(1\right)},p_{3}^{\left(1\right)}\right\}$, $p_{4}^{\left(1\right)},p_{5}^{\left(1\right)},p_{6}^{\left(1\right)}\}$, $\left\{p_{7}^{\left(1\right)},p_{8}^{\left(1\right)},p_{9}^{\left(1\right)}\right\}$, and $\left\{p_{10}^{\left(1\right)},p_{11}^{\left(1\right)},p_{12}^{\left(1\right)}\right\}$ are highly correlated, *ρ *= 0.94, by the generating signal (3). In the diagrams, proteins $p_{1}^{\left(1\right)},\ldots ,p_{12}^{\left(1\right)}$ are represented by *A, . . . , L *for ease of reading. In all five figures, red, blue and brown edges signify edge probabilities of greater than 0.9, between 0.8 and 0.9, and from 0.5 to 0.8, respectively. The composite has high posterior probabilities for all edges with at least moderate network partial correlations. One of the three reps, $R_{2}^{\left(1\right)}$, misidentifies a small number of edges.

Figure 1 displays the moderate to high edge posterior probabilities of Study1. From Equation (4), the generating partial correlations between $p_{1}^{\left(1\right)}$ and $p_{2}^{\left(1\right)}$ and between $p_{2}^{\left(1\right)}$ and $p_{3}^{\left(1\right)}$ equal 0.685, and the generating partial correlation for $p_{1}^{\left(1\right)}$ and $p_{3}^{\left(1\right)}$ is zero. It is no coincidence that the edge posterior probabilities for $p_{1}^{\left(1\right)}-p_{2}^{\left(1\right)}$ and $p_{2}^{\left(1\right)}-p_{3}^{\left(1\right)}$ are no lower than 0.9139 in any one of the reps and the composite. Furthermore, the edge probabilities for $p_{1}^{\left(1\right)}-p_{3}^{\left(1\right)}$ do not exceed 0.0501. However, as seen in Table 1, the triple connectivity probabilities for proteins $p_{1}^{\left(1\right)}$, $p_{2}^{\left(1\right)}$, and $p_{3}^{\left(1\right)}$ remain extremely high.

Consider a particular one of the generating 3 *× *3 blocks in this first simulation, say based on the ordered proteins: *A*, *B*, and *C*. It has high moderate Pearson correlations between all of its protein pairs. There is also substantial partial correlation between two adjacency proteins, namely between *A *and *B*, and also between *B *and *C*. However, there is zero partial correlation between the non-adjacent proteins *A *and *C*. In other words, for a fixed value of *B*, there is no correlation between *A *and *C*. It is informative to compare this setting to the biological setting where a parent protein A^* ^has a causal influence on a child protein B^* ^which has a causal influence on a grandchild protein C^*^. However, for a fixed result for B^*^, A^* ^has no influence on C^*^. Hence, there is no partial causal influence between A^* ^and C^*^.

The estimation of partial correlation for sparse biological data is accomplished through the Lasso, adaptive Lasso and Ridge techniques [6,19-26]. Table 2 shows sample partial correlation estimates for $R_{1}^{\left(1\right)}$ using Lasso and adaptive Lasso (entries above and below the main diagonal, respectively). These two sets of partial correlation estimates for $R_{1}^{\left(1\right)}$ strongly reflect the true generating partial correlation. A sample partial correlation estimate for $R_{1}^{\left(1\right)}$ based on Ridge is shown in Table 3. This estimate also captures the true underlying partial correlation, though not as cleanly. There is much less zero partial correlation from the Ridge technique than there is from the Lasso and adaptive Lasso techniques. Computationally, the Ridge technique involves a quadratic penalty parameter on slope magnitude, while the Lasso and adaptive Lasso techniques adopt a linear penalty.

**Table 2:** Lasso and adaptive Lasso partial correlation estimates for $R_{1}^{\left(1\right)}$

	$p_{1}^{\left(1\right)}$	$p_{2}^{\left(1\right)}$	$p_{3}^{\left(1\right)}$	$p_{4}^{\left(1\right)}$	$p_{5}^{\left(1\right)}$	$p_{6}^{\left(1\right)}$	$p_{7}^{\left(1\right)}$	$p_{8}^{\left(1\right)}$	$p_{9}^{\left(1\right)}$	$p_{10}^{\left(1\right)}$	$p_{11}^{\left(1\right)}$	$p_{12}^{\left(1\right)}$
$p_{1}^{\left(1\right)}$	**1.00**	0.77	0.00	0.00	0.00	0.00	0.00	0.00	0.00	0.00	0.00	0.00
$p_{2}^{\left(1\right)}$	0.84	**1.00**	0.56	0.00	0.00	0.00	0.00	0.00	0.00	0.00	0.00	0.00
$p_{3}^{\left(1\right)}$	0.00	0.48	**1.00**	0.00	0.00	0.00	0.00	0.00	0.00	0.00	0.00	0.00
$p_{4}^{\left(1\right)}$	0.00	0.00	0.00	**1.00**	0.76	0.00	0.00	0.00	0.00	0.00	0.00	0.00
$p_{5}^{\left(1\right)}$	0.00	0.00	0.00	0.58	**1.00**	0.00	0.00	0.00	0.00	0.00	0.00	0.00
$p_{6}^{\left(1\right)}$	0.00	0.00	0.00	0.00	0.42	**1.00**	0.00	0.00	0.00	0.00	0.00	0.00
$p_{7}^{\left(1\right)}$	0.00	0.00	0.00	0.00	0.00	0.00	**1.00**	0.96	0.00	0.00	0.00	0.00
$p_{8}^{\left(1\right)}$	0.00	0.00	0.00	0.00	0.00	0.00	0.70	**1.00**	0.00	0.00	0.00	0.00
$p_{9}^{\left(1\right)}$	0.00	0.00	0.15	0.00	0.00	0.00	0.32	0.28	**1.00**	0.00	0.00	0.00
$p_{10}^{\left(1\right)}$	0.00	-0.03	0.00	0.00	0.00	0.00	0.00	0.00	0.00	**1.00**	0.89	0.27
$p_{11}^{\left(1\right)}$	0.00	0.00	0.00	0.00	0.00	0.00	0.01	0.00	0.00	0.63	**1.00**	0.00
$p_{12}^{\left(1\right)}$	0.00	0.00	0.06	0.00	0.00	0.00	0.01	0.00	0.00	0.50	0.28	**1.00**

Sample partial correlation estimates for replicate $R_{1}^{\left(1\right)}$ are computed using both the Lasso and adaptive Lasso methods. These estimates are shown below and above the main diagonal, respectively. These replicate partial correlation estimates reflect the generating partial correlations, though not exactly.

**Table 3:** Ridge partial correlation estimates for $R_{1}^{\left(1\right)}$

	$p_{1}^{\left(1\right)}$	$p_{2}^{\left(1\right)}$	$p_{3}^{\left(1\right)}$	$p_{4}^{\left(1\right)}$	$p_{5}^{\left(1\right)}$	$p_{6}^{\left(1\right)}$	$p_{7}^{\left(1\right)}$	$p_{8}^{\left(1\right)}$	$p_{9}^{\left(1\right)}$	$p_{10}^{\left(1\right)}$	$p_{11}^{\left(1\right)}$
**$p_{2}^{\left(1\right)}$**	0.75	**1.00**									
**$p_{3}^{\left(1\right)}$**	0.10	0.30	**1.00**								
**$p_{4}^{\left(1\right)}$**	0.49	-0.49	0.00	**1.00**							
**$p_{5}^{\left(1\right)}$**	0.00	0.06	0.13	0.54	**1.00**						
**$p_{6}^{\left(1\right)}$**	-0.16	0.14	0.06	0.31	0.31	**1.00**					
**$p_{7}^{\left(1\right)}$**	0.14	0.05	0.00	0.02	-0.07	0.00	**1.00**				
**$p_{8}^{\left(1\right)}$**	-0.16	0.08	0.07	0.11	-0.05	-0.08	0.49	**1.00**			
**$p_{9}^{\left(1\right)}$**	0.00	-0.04	0.16	0.13	-0.07	0.00	0.43	0.39	**1.00**		
**$p_{10}^{\left(1\right)}$**	0.07	-0.09	0.00	0.18	0.00	0.00	-0.20	-0.05	-0.04	**1.00**	
**$p_{11}^{\left(1\right)}$**	0.21	0.03	-0.08	-0.16	0.00	0.06	0.38	-0.00	-0.09	0.50	**1.00**
**$p_{12}^{\left(1\right)}$**	-0.21	-0.05	0.09	-0.22	0.11	0.05	-0.05	0.00	0.19	0.54	0.40

Sample partial correlation estimates for $R_{1}^{\left(1\right)}$ are computed using the Ridge technique.

In Table 4, the protein to protein sample Pearson correlations of $R_{1}^{\left(1\right)}$ are shown. Note that, as expected from the generator, $p_{1}^{\left(1\right)}-p_{3}^{\left(1\right)}$ has high sample Pearson correlation, despite its near zero sample partial correlation estimates.

**Table 4:** Pearson correlations for $R_{1}^{\left(1\right)}$

	$p_{1}^{\left(1\right)}$	$p_{2}^{\left(1\right)}$	$p_{3}^{\left(1\right)}$	$p_{4}^{\left(1\right)}$	$p_{5}^{\left(1\right)}$	$p_{6}^{\left(1\right)}$	$p_{7}^{\left(1\right)}$	$p_{8}^{\left(1\right)}$	$p_{9}^{\left(1\right)}$	$p_{10}^{\left(1\right)}$	$p_{11}^{\left(1\right)}$
**$p_{1}^{\left(1\right)}$**	0.94	**1.00**									
**$p_{2}^{\left(1\right)}$**	0.82	0.92	**1.00**								
**$p_{3}^{\left(1\right)}$**	0.16	0.14	0.31	**1.00**							
**$p_{4}^{\left(1\right)}$**	0.18	0.22	0.39	0.90	**1.00**						
**$p_{5}^{\left(1\right)}$**	0.07	0.19	0.31	0.78	0.86	**1.00**					
**$p_{6}^{\left(1\right)}$**	0.55	0.64	0.61	-0.06	-0.18	-0.09	**1.00**				
**$p_{7}^{\left(1\right)}$**	0.51	0.61	0.62	-0.05	-0.16	-0.12	0.98	**1.00**			
**$p_{8}^{\left(1\right)}$**	0.54	0.64	0.69	0.03	-0.07	-0.01	0.97	0.97	**1.00**		
**$p_{9}^{\left(1\right)}$**	-0.20	-0.18	-0.08	-0.09	0.00	0.12	0.02	-0.02	0.01	**1.00**	
**$p_{10}^{\left(1\right)}$**	-0.11	0.08	-0.02	-0.16	-0.06	0.08	0.17	0.10	0.13	0.97	**1.00**
**$p_{11}^{\left(1\right)}$**	-0.06	0.01	0.15	-0.01	0.09	0.22	0.26	0.21	0.27	0.95	0.95

Sample Pearson correlations for $R_{1}^{\left(1\right)}$.

The second simulation study consists of *t *= 5 measurements of 9 proteins, $p_{1}^{\left(2\right)},\ldots ,p_{9}^{\left(2\right)}$. The replicate data is generated from a signal giving correlation intensity *ρ *= 0.9 to each of the two triple blocks, $\left\{p_{1}^{\left(2\right)},p_{2}^{\left(2\right)},p_{3}^{\left(2\right)}\right\}$ and $\left\{p_{7}^{\left(2\right)},p_{8}^{\left(2\right)},p_{9}^{\left(2\right)}\right\}$, as well as the block of two proteins $\left\{p_{4}^{\left(2\right)},p_{5}^{\left(2\right)}\right\}$. All other pairs of proteins are assigned zero correlation. Note that protein $p_{6}^{\left(2\right)}$has zero signal correlation to all other proteins.

**Figure 2:**
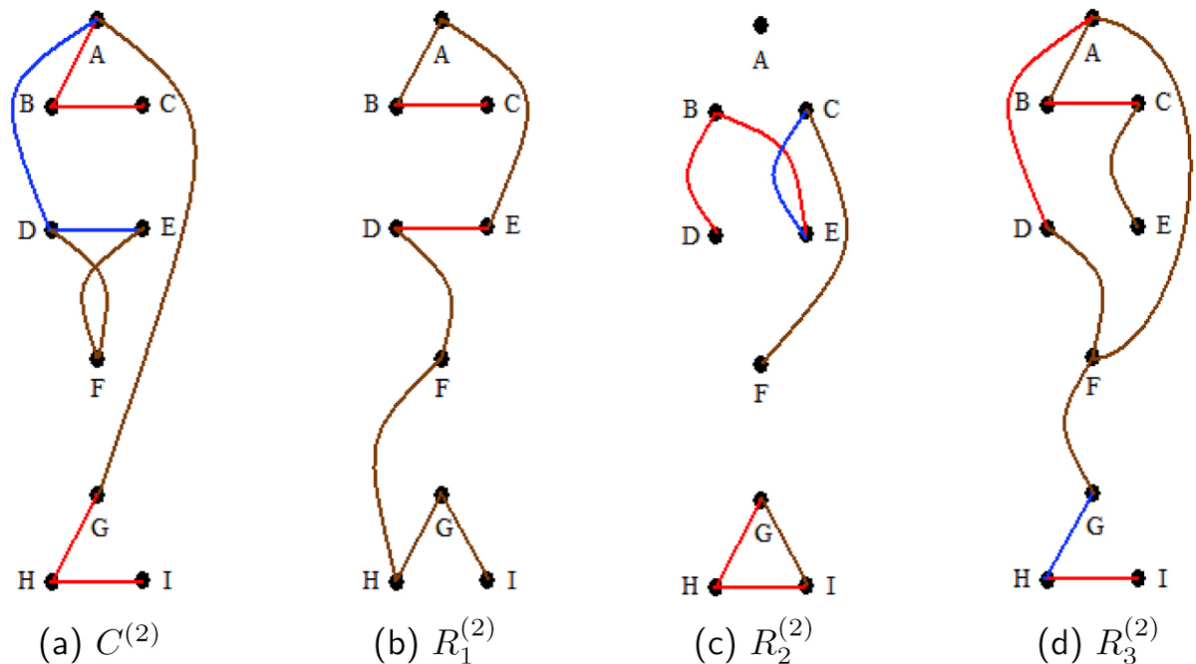
**Study 2 undirected edge posterior probabilities**. For the second study, (a), (b), (c) and (d) are representations of the undirected edge posterior probabilities. These are computed for the composite of all the replicates, *C*^(2)^, and for the individual replicates, $R_{1}^{\left(2\right)}$, $R_{2}^{\left(2\right)}$ and $R_{3}^{\left(2\right)}$. Blocks of vertices $\left\{p_{1}^{\left(2\right)},p_{2}^{\left(2\right)},p_{3}^{\left(2\right)}\right\}$, $\left\{p_{4}^{\left(2\right)},p_{5}^{\left(2\right)}\right\}$, $\left\{p_{6}^{\left(2\right)}\right\}$, and $\left\{p_{7}^{\left(2\right)},p_{8}^{\left(2\right)},p_{9}^{\left(2\right)}\right\}$ are highly correlated, *ρ *= 0.9, by the generating signal (3). In the diagrams proteins $p_{1}^{\left(2\right)},\ldots ,p_{9}^{\left(2\right)}$ are represented by *A, . . . , I *for ease of reading. As in the first study, the composite has high posterior probabilities for all edges with at least moderate network partial correlations. Overall, the smaller blocks were not identified as well as the triple blocks. The signal was not identified as well for $R_{1}^{\left(2\right)}$.

The triple and double probabilities also are examined. Table 5 depicts the analysis of these posterior probabilities. An interesting result is the low $\left\{p_{1}^{\left(2\right)},p_{2}^{\left(2\right)},p_{3}^{\left(2\right)}\right\}$ triple probability of 0.0416 for $R_{2}^{\left(2\right)}$. This is due to $R_{2}^{\left(2\right)}$ having low double probabilities amongst proteins $p_{1}^{\left(2\right)},p_{2}^{\left(2\right)}$ and $p_{3}^{\left(2\right)}$ (see Figure 2). For $R_{2}^{\left(2\right)}$, the two edges $p_{1}^{\left(2\right)}-p_{3}^{\left(2\right)}$ and $p_{2}^{\left(2\right)}-p_{3}^{\left(2\right)}$ have probabilities of 0.0880 and 0.1006, respectively. The sample Lasso and adaptive Lasso partial correlations estimates in $R_{2}^{\left(2\right)}$ for these 2 edges are zero. Our composite analysis of *C*^(2) ^as well as the individual rep analysis of $R_{1}^{\left(2\right)}$ are successful in recognizing the generating signal, whereas individual rep analyses of $R_{2}^{\left(2\right)}$ and $R_{3}^{\left(2\right)}$ do not fare as well. Many of these $R_{2}^{\left(2\right)}$ and $R_{3}^{\left(2\right)}$ deviations from signal are associated with corresponding deviations of their sample partial correlations from the signal, which is most likely caused by the small sample size of *t *= 5.

**Table 5:** Study 2 posterior probabilities

	C^(1)^	$R_{1}^{\left(1\right)}$	$R_{2}^{\left(1\right)}$	$R_{3}^{\left(1\right)}$
$p_{1}^{\left(2\right)}\;-p_{2}^{\left(2\right)}\;-p_{3}^{\left(2\right)}$	1.0	0.9902	0.0416	0.7189
$p_{7}^{\left(2\right)}\;-p_{8}^{\left(2\right)}\;-p_{9}^{\left(2\right)}$	1.0	1.0	0.9397	1.0
TEP Avg	0.1482	0.1053	0.1680	0.1440
TLFP Avg	0.0185	0.0212	0.0821	0.0377
$p_{4}^{\left(2\right)}\;-p_{5}^{\left(2\right)}\;$	0.8624	0.9974	0.0659	0.3481
DFP Avg	0.1943	0.1493	0.2612	0.2174
DLEP Avg	0.0488	0.0536	0.1692	0.0996

For the second study, the posterior connectivity probabilities for the three signal connected blocks (*ρ *= 0.9) are shown on rows 1, 2 and 5. The posterior probabilities averages are shown for all false triple and double possibilities on rows 3 and 4, and 6 and 7, respectively.

The third study contains 10 measurements of 10 proteins, $p_{1}^{\left(3\right)},\ldots ,p_{10}^{\left(3\right)}$. An underlying signal is generated with correlation intensity *ρ *= 0.94 for the two quadruple blocks of vertices $\left\{p_{1}^{\left(3\right)},\ldots ,p_{4}^{\left(3\right)}\right\}$ and $\left\{p_{5}^{\left(3\right)},\ldots ,p_{8}^{\left(3\right)}\right\}$ as well as the double block $\left\{p_{9}^{\left(3\right)},p_{10}^{\left(3\right)}\right\}$.

High signal correlations between proteins yield high sample Pearson correlations (ranging from 0.73 to 0.99) within the reps. Additionally, the zero signal correlation pairs yield low sample Pearson correlations, ranging from -0.13 to 0.40.

The quadruple and double connectivity probabilities are shown in Table 6. For signal blocks in $C_{}^{\left(3\right)}$, $R_{1}^{\left(3\right)}$, $R_{2}^{\left(3\right)}$, and $R_{3}^{\left(3\right)}$, the computed quadruple and double probabilities are all 1.0. This indicates each one of the top undirected graphs has a connection within $\left\{p_{1}^{\left(3\right)},\ldots ,p_{4}^{\left(3\right)}\right\}$, $\left\{p_{5}^{\left(3\right)},\ldots ,p_{8}^{\left(3\right)}\right\}$, and $\left\{p_{9}^{\left(3\right)},p_{10}^{\left(3\right)}\right\}$. The average connectivity probabilities for non-blocks is low, with none exceeding 0.1203 in any of the models.

**Table 6:** Study 3 posterior probabilities

	C^(3)^	$R_{1}^{\left(3\right)}$	$R_{2}^{\left(3\right)}$	$R_{3}^{\left(3\right)}$
$p_{1}^{\left(3\right)}\;-p_{2}^{\left(3\right)}\;-p_{3}^{\left(3\right)}-p_{4}^{\left(3\right)}$	1.0	1.0	1.0	1.0
$p_{5}^{\left(3\right)}\;-p_{6}^{\left(3\right)}\;-p_{7}^{\left(3\right)}-p_{8}^{\left(3\right)}$	1.0	1.0	1.0	1.0
QFP Avg	0.0611	0.0683	0.0809	0.0459
QLFP Avg	0.0035	0.0038	0.0059	0.0008
$p_{9}^{\left(3\right)}\;-p_{10}^{\left(3\right)}\;$	1.0	1.0	1.0	1.0
DFP Avg	0.094	0.1203	0.1044	0.0751
DLEP Avg	0.0232	0.0094	0.0144	0.0045

For the third study, the posterior connectivity probabilities for the three signal connected blocks, *ρ *= 0.94, are shown on rows 1, 2 and 5. The posterior probabilities averages for all non-signal generated quadruple and double possibilities are shown on rows 3 and 4, and 6 and 7, respectively.

**Figure 3:**
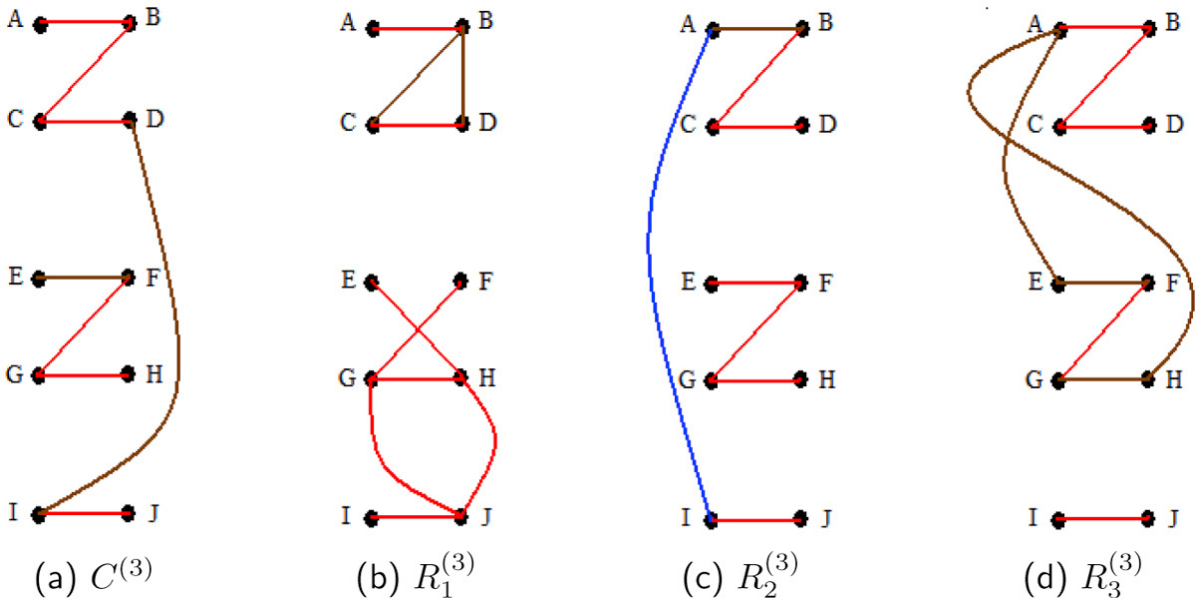
**Study 3 undirected edge posterior probabilities**. For the third study, (a), (b), (c) and (d) are representations of the undirected edge posterior probabilities. These are computed for the composite of all the replicates, *C*^(3)^, and for the individual replicates, $R_{1}^{\left(3\right)}$, $R_{2}^{\left(3\right)}$ and $R_{3}^{\left(3\right)}$. Blocks of vertices $\left\{p_{1}^{\left(3\right)},p_{2}^{\left(3\right)},p_{3}^{\left(3\right)},p_{4}^{\left(3\right)}\right\}$, $\left\{p_{5}^{\left(3\right)},p_{6}^{\left(3\right)},p_{7}^{\left(3\right)},p_{8}^{\left(3\right)}\right\}$, and $\left\{p_{9}^{\left(3\right)},p_{10}^{\left(3\right)}\right\}$ are highly correlated, *ρ *= 0.94, by the generating signal (3). In the diagrams, proteins $p_{1}^{\left(3\right)},\ldots ,p_{10}^{\left(3\right)}$ are represented by *A, . . . , J *for ease of reading. The composite has high posterior probabilities for all edges with at least moderate partial correlations. The composite analysis outperforms at least two of the single replicate analyses.

For the quadruple block of proteins, $\left\{p_{1}^{\left(3\right)},p_{2}^{\left(3\right)},p_{3}^{\left(3\right)},p_{4}^{\left(3\right)}\right\}$, signal partial correlations between $p_{1}^{\left(3\right)}$ and $p_{2}^{\left(3\right)}$, and between $p_{3}^{\left(3\right)}$ and $p_{4}^{\left(3\right)}$ equal 0.685, and the signal partial correlation between $p_{2}^{\left(3\right)}$ and $p_{3}^{\left(3\right)}$ is 0.499. All other combinations of two proteins in this block have a signal partial correlation of zero. As might be expected, the edge posterior probabilities for $p_{1}^{\left(3\right)}-p_{2}^{\left(3\right)}$, $p_{3}^{\left(3\right)}-p_{4}^{\left(3\right)}$, and $p_{2}^{\left(3\right)}-p_{3}^{\left(3\right)}$ are high, averaging 0.95 among the three reps and the composite. All other edge probabilities within this block have an average of 0.105 (see Figure 3). Nevertheless, each of the probabilities that all four proteins connect is 1.0 in all reps and the composite (view Table 6).

**Figure 4:**
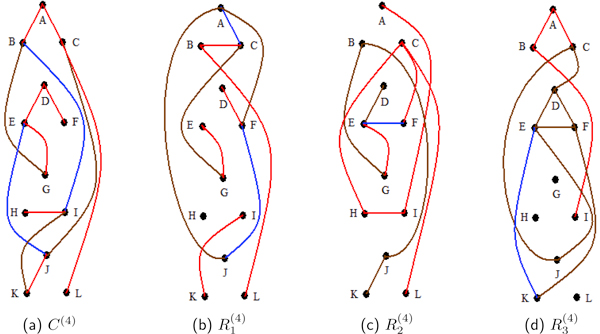
**Study 4 undirected edge posterior probabilities**. For the fourth study, (a), (b), (c) and (d) are representations of the undirected edge posterior probabilities. These are computed for the composite of all the replicates, *C*^(4)^, and for the individual replicates, $R_{1}^{\left(4\right)}$, $R_{2}^{\left(4\right)}$ and $R_{3}^{\left(4\right)}$. Blocks of vertices $\left\{p_{1}^{\left(4\right)},p_{2}^{\left(4\right)},p_{3}^{\left(4\right)}\right\}$, $\left\{p_{4}^{\left(4\right)},p_{5}^{\left(4\right)},p_{6}^{\left(4\right)}\right\}$, $\left\{p_{7}^{\left(4\right)},p_{8}^{\left(4\right)},p_{9}^{\left(4\right)}\right\}$, and $\left\{p_{10}^{\left(4\right)},p_{11}^{\left(4\right)},p_{12}^{\left(4\right)}\right\}$ are signal correlated with *ρ *values of 0.7, 0.6, 0.6 and 0.5, respectively for each block (3). In the diagrams proteins $p_{1}^{\left(4\right)},\ldots ,p_{12}^{\left(4\right)}$ are represented by *A, . . . , L *for ease of reading. With this study's smaller partial correlations within blocks, the network signal is not recovered as well as in the three previous studies. Still, the composite model outperforms each of the individual replicates' models.

The fourth study complements the first study but with lower and decreasing correlation intensities among the 4 blocks of three proteins. The assigned *ρ *values to the four blocks of three proteins are 0.7, 0.6, 0.6 and 0.5, respectively. As in the previous three studies, average sample correlations between proteins in different blocks remain low throughout the reps, ranging from -0.0329 to 0.1159. The sample correlations between proteins that are signal correlated within blocks are representative of the signal correlations. The exception occurs in $R_{2}^{\left(4\right)}$, where $R_{2}^{\left(4\right)}$ has relatively low within block sample correlations ranging from *-*0.1141 to 0.7817. This influenced its signal inconsistencies in edge probabilities (refer to Figure 4). In general, triples associated with lower generating *ρ *values receive lower and more inconsistent correlations, which also speak to some edge probability inconsistencies in Figure 4.

The triple connectivity probabilities can be seen in Table 7. With lower signal correlations among the triples, the triple connectivity posterior probabilities are not quite as strong as in the previous studies. However, the composite performs at least as well if not better than each of the individual replicates.

**Table 7:** Study 4 posterior probabilities

	C^(4)^	$R_{1}^{\left(4\right)}$	$R_{2}^{\left(4\right)}$	$R_{3}^{\left(4\right)}$
$p_{1}^{\left(4\right)}\;-p_{2}^{\left(4\right)}\;-p_{3}^{\left(4\right)}$	1.0	1.0	0.0	1.0
$p_{4}^{\left(4\right)}\;-p_{5}^{\left(4\right)}\;-p_{6}^{\left(4\right)}$	0.9874	0.3109	0.6007	0.8037
$p_{7}^{\left(4\right)}\;-p_{8}^{\left(4\right)}\;-p_{9}^{\left(4\right)}$	0.0455	0.0021	0.0095	0.0106
$p_{10}^{\left(4\right)}\;-p_{11}^{\left(4\right)}\;-p_{12}^{\left(4\right)}$	0.3655	0.0038	0.1190	0.0117
TFP Avg	0.0853	0.0467	0.0692	0.0700
TLFP Avg	0.0043	0.0018	0.0046	0.0068

For the fourth study, the posterior connectivity probabilities are shown for the four signal connected blocks with *ρ *values of 0.7, 0.6, 0.6, and 0.5, respectively. For the composite models and the single rep models, all these are quite high. The last two rows indicate the posterior probability averages over all other non-block threesome of proteins; these averages are very low.

**Figure 5:**
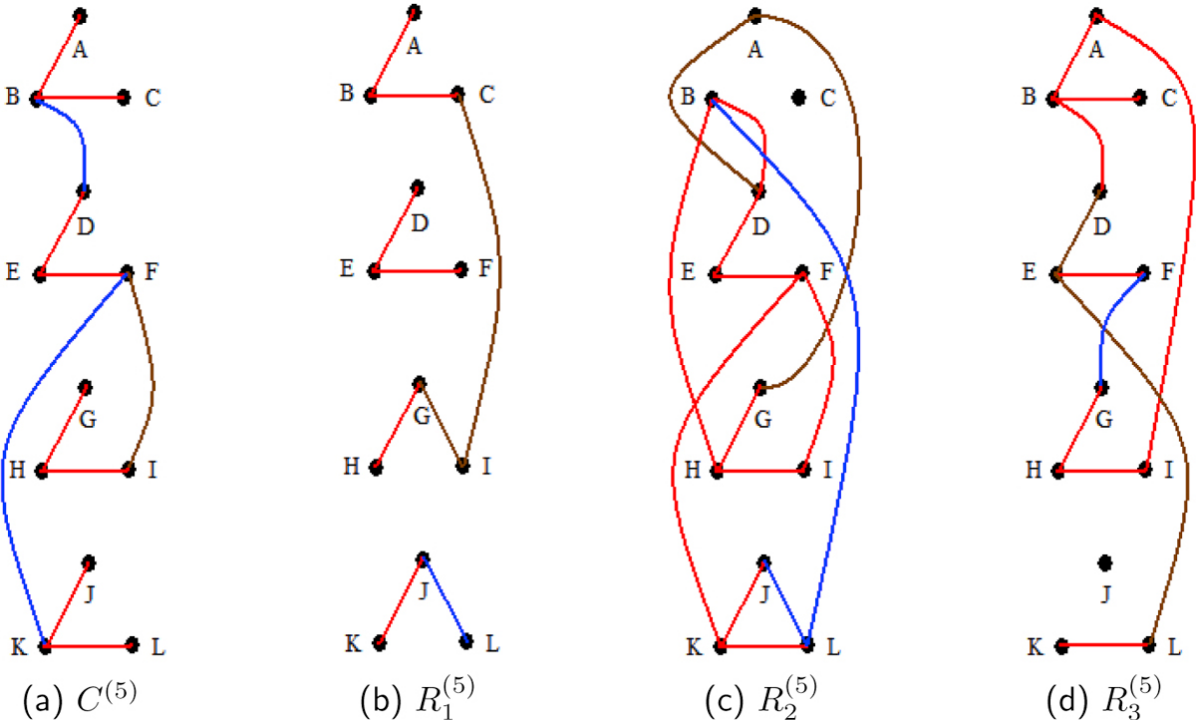
**Study 5 undirected edge posterior probabilities**. For the fifth study, (a), (b), (c) and (d) are representations of the undirected edge posterior probabilities. These are computed for the composite of all the replicates, *C*^(5)^, and for the individual replicates, $R_{1}^{\left(5\right)}$, $R_{2}^{\left(5\right)}$ and $R_{3}^{\left(5\right)}$. Blocks of vertices $\left\{p_{1}^{\left(5\right)},p_{2}^{\left(5\right)},p_{3}^{\left(5\right)}\right\}$, $\left\{p_{4}^{\left(5\right)},p_{5}^{\left(5\right)},p_{6}^{\left(5\right)}\right\}$, $\left\{p_{7}^{\left(5\right)},p_{8}^{\left(5\right)},p_{9}^{\left(5\right)}\right\}$ and $\left\{p_{10}^{\left(5\right)},p_{11}^{\left(5\right)},p_{12}^{\left(5\right)}\right\}$ are signal correlated in $R_{1}^{\left(5\right)}$ are with *ρ *= 0.9, in $R_{2}^{\left(5\right)}$ with *ρ *= 0.82, and in $R_{3}^{\left(5\right)}$ with *ρ *= 0.7 (3). In the diagrams proteins $p_{1}^{\left(5\right)},\ldots ,p_{12}^{\left(5\right)}$ are represented by *A, . . . , L *for ease of reading. The composite has high posterior probabilities for all edges with at least moderate network partial correlations. The composite model outperforms each of the individual replicates' models.

The fifth study uses the signal topology of the first and fourth studies. However, the first replicate has correlation intensity of *ρ *= 0.9, the second replicate has *ρ *= 0.82 and the third replicate has *ρ *= 0.7. In this study the generating signal is not as strong as the signals in the first three studies. The block correlations for each replicate are derived from the *ρ *value assigned to each rep. As expected, $R_{1}^{\left(5\right)}$ has the highest sample correlations among triple signal correlated proteins, followed by $R_{2}^{\left(5\right)}$ and lastly $R_{3}^{\left(5\right)}$. All have small sample correlation averages among signal zero correlated proteins. The edge probability diagrams (Figure 5) are symbolic of these results.

Table 8 shows triple connectivity posterior probabilities. Analyses for *C*^(5) ^and $R_{1}^{\left(5\right)}$ show triple connectivity probabilities of 1.0 for all blocks, while the single rep analysis for $R_{2}^{\left(5\right)}$ does not recognize signal in $\left\{p_{1}^{\left(5\right)},p_{2}^{\left(5\right)},p_{3}^{\left(5\right)}\right\}$ (see Figure 5). The analysis for $R_{3}^{\left(5\right)}$, which was generated with the lowest correlation intensity, does not recognize the signal as well as those of $R_{1}^{\left(5\right)}$ and *C*^(5)^.

**Table 8:** Study 5 posterior probabilities

	C^(5)^	$R_{1}^{\left(5\right)}$	$R_{2}^{\left(5\right)}$	$R_{3}^{\left(5\right)}$
$p_{1}^{\left(5\right)}\;-p_{2}^{\left(5\right)}\;-p_{3}^{\left(5\right)}$	1.0	1.0	0.0016	1.0
$p_{4}^{\left(5\right)}\;-p_{5}^{\left(5\right)}\;-p_{6}^{\left(5\right)}$	1.0	1.0	0.9874	0.9819
$p_{7}^{\left(4\right)}\;-p_{8}^{\left(4\right)}\;-p_{9}^{\left(4\right)}$	1.0	1.0	1.0	1.0
$p_{10}^{\left(5\right)}\;-p_{11}^{\left(5\right)}\;-p_{12}^{\left(5\right)}$	1.0	1.0	1.0	0.3477
TFP Avg	0.0643	0.0418	0.0854	0.0613
TLFP Avg	0.0019	0.0023	0.0017	0.0017

For the fifth study, the posterior connectivity probabilities for the four signal connected blocks are shown, where $R_{1}^{\left(5\right)},R_{2}^{\left(5\right)},R_{3}^{\left(5\right)}$are generated with three different *ρ *values, 0.9, 0.82 and 0.7, respectively. For the composite model and the single rep models, all of these are very high. The last two rows indicate the very low average posterior probability over all non-signal threesomes of vertices.

### ROC analysis of posterior probabilities

**Figure 6:**
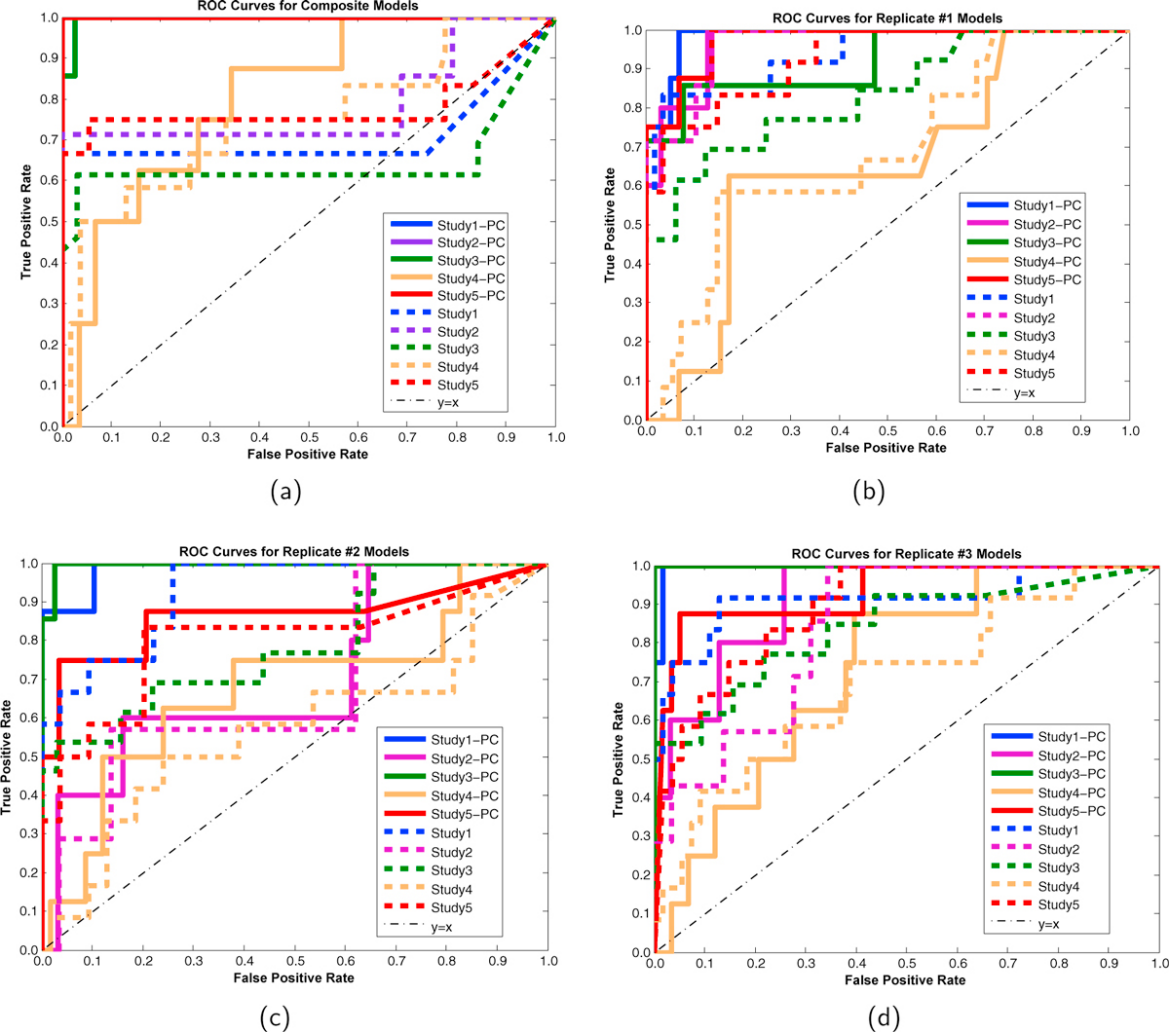
**ROC curves of edge posterior probabilities**. The receiver operating characteristic curves (ROC) of edge posterior probabilities are shown for the composite and replicate models in each of the five studies. In each graph, the solid and dashed lines are the ROCs corresponding to the generating partial correlations and generating Pearson correlations, respectively. On each ROC curve, the ROC points correspond to the decreasing sequence of edge posterior probabilities. The partial correlation ROC curves are better than (above) the corresponding Pearson correlation curves. The composite's partial correlation ROC curves outperform the corresponding curves for each of the individual replications.

The receiver operating characteristic (ROC) curves [27] for the composite and the individual replicates from each of the five studies are shown in Figure 6. The ROC (*x*, *y*) coordinates are generated by the decreasing sequence of edge posterior probability *cutoffs *(i.e. lower limits for classifying positive edges). The *y*-coordinate, the true positive rate (TPR), is the fraction of signal edges that are classified as positive edges. The *x*-coordinate, the false positive rate (FPR), is the fraction of signal non-edges that are classified as positive edges. In each of Figures 6(a)-6(d), comparisons are made between ROC curves whose signal edges are determined by non-zero Pearson correlation (ranging from 0.250 to 0.940) versus those whose signal edges are determined by non-zero partial correlation (ranging from 0.447 to 0.685). The signal partial correlation ROC curves tend to be above and to the left of the signal Pearson correlation ROC curves. This represents the algorithm's ability to identify, with higher posterior probability, signal partial correlation edges over signal Pearson correlation edges.

As can be seen in covariance block (3) and partial correlation matrix (4), there are more signal Pearson correlation edges than signal partial correlation edges. For the ROC curves based on partial correlation edges, there are only two edges within a triple block, and each has a relatively high posterior probability. This leads to their ROC curves increasing at a faster rate than those based on Pearson correlation edges. Overall, the signal non-zero Pearson correlation edges have lower posterior probabilities than do the signal non-zero partial correlation edges. In addition, the composite ROC curves tend to be to the upper left of their corresponding individual replication ROC curves. This corresponds to higher posterior probabilities for the true signal edges under composite analysis than under most individual rep analyses.

## Conclusions

Structured Bayesian posterior probabilities are developed for network features based on multiple sparse time-course data sets. This methodology allows for the incorporation of data sets with varying degree of experimental variability. For our simulations, the multiple rep composite method performs well in uncovering strong network signals. The composite method does better than a single rep method in uncovering moderate network signals. The composite method assigns high posterior probability to edges with at least moderate network partial correlation, while it assigns moderate to small posterior probabilities to edges with 0.0 network partial correlation.

Composite ROC curves based on system non-zero partial correlation (solid lines in Figure 6(a)) have small area above them which signifies that our composite method provides excellent detection of edges having partial correlation.

The five simulation studies span a range of network situations. The first three studies examine networks consisting of block subnetworks with high signal correlations within blocks. These blocks are of varying sizes. The composite method is more successful in identifying blocks of three or four proteins, rather than smaller blocks. For study four, blocks with moderate, rather than high, signal correlation within the blocks are examined. The composite method does not perform as well for these blocks but it does outperform the single rep method for all study four's subnetworks. In the fifth study, the different reps have varying degree of experimental variability. Still, the composite method recognizes the network's signals with high posterior probabilities.

The multiple rep method utilizes independent empirical priors acting on independent reps. Thus, as suggested by the fifth study, this method can be used even if there are major fixed, non-random differences between the reps. Each rep still contributes information about the network structure. This likelihood based methodology automatically weights the reps in the sense that reps having more experimental variability will receive less weight in determining the subnetworks which have highest posterior probability.

The computation of posterior probabilities lends itself towards the identification of various network features. These features can correspond to connected subgraphs in the interaction network. With experimental data, where the goal is to discover the generating signal, searching for high probability features is quite valuable.

If there are strictly random differences between the reps, it may be useful to employ a hierarchical structure (e.g. assuming that parent-child regression slopes for one rep come from the same distributions as do those from another rep). This approach would involve substantially more complex Bayes factors, and thus would be more computationally intensive. We are currently developing methodology for this setting.

## List of abbreviations

BIC: Bayesian Information Criterion; DAG: directed acyclic graph; DFP: double block false positive; DLFP: double block log odds based false positive; FPR: false positive rate; QFP: quadruple block false positive; QLFP: quadruple block log odds based false positive; rep: replicate; ROC: receiver operating characteristic; TFP: triple block false positive; TLFP: triple block log odds based false positive; TPR: true positive rate.

## Competing interests

The authors declare that they have no competing interests.

## Authors' contributions

All three authors contributed equally in developing the ideas, running and analyzing simulated data, assessing the quality of the models, and writing the paper.
